# Dose-dependent effects of gamma radiation sterilization on the collagen matrix of human cortical bone allograft and its influence on fatigue crack propagation resistance

**DOI:** 10.1007/s10561-024-10135-2

**Published:** 2024-05-15

**Authors:** Dylan B. Crocker, Thomas M. Hering, Ozan Akkus, Megan E. Oest, Clare M. Rimnac

**Affiliations:** 1 Department of Mechanical and Aerospace Engineering, Case Western Reserve University, Cleveland, OH USA; 2 Department of Biomedical Engineering, Case Western Reserve University, Cleveland, OH USA; 3 Department of Orthopedic Surgery, SUNY Upstate Medical University, Syracuse, NY USA

**Keywords:** Bone, Radiation, Collagen, Crosslinking, Allografts

## Abstract

Fatigue crack propagation resistance and high-cycle S–N fatigue life of cortical bone allograft tissue are both negatively impacted in a radiation dose-dependent manner from 0 to 25 kGy. The standard radiation sterilization dose of 25–35 kGy has been shown to induce cleavage of collagen molecules into smaller peptides and accumulation of stable crosslinks within the collagen matrix, suggesting that these mechanisms may influence radiation-induced losses in cyclic fracture resistance. The objective of this study was to determine the radiation dose-dependency of collagen chain fragmentation and crosslink accumulation within the dose range of 0–25 kGy. Previously, cortical bone compact tension specimens from two donor femoral pairs were divided into four treatment groups (0 kGy, 10 kGy, 17.5 kGy, and 25 kGy) and underwent cyclic loading fatigue crack propagation testing. Following fatigue testing, collagen was isolated from one compact tension specimen in each treatment group from both donors. Radiation-induced collagen chain fragmentation was assessed using SDS-PAGE (n = 5), and accumulation of pentosidine, pyridinoline, and non-specific advanced glycation end products were assessed using a fluorometric assay (n = 4). Collagen chain fragmentation increased progressively in a dose-dependent manner (*p* < 0.001). Crosslink accumulation at all radiation dose levels increased relative to the 0 kGy control but did not demonstrate dose-dependency (*p* < 0.001). Taken together with our previous findings on fatigue crack propagation behavior, these data suggest that while collagen crosslink accumulation may contribute to reduced notched fatigue behavior with irradiation, dose-dependent losses in fatigue crack propagation resistance are mainly influenced by radiation-induced chain fragmentation.

## Introduction

Structural cortical bone allografts are commonly used in reconstruction of large bone defects resulting from trauma, cancer, revision joint arthroplasty, and non-union following fracture healing (Dincel 2018; Singh et al. 2016; Stok et al. 2011; Archunan and Petronis 2021). Approximately 10% of all skeletal reconstructive surgeries involve the use of bone allografts (Stok et al. 2011). However, allografts carry the risk of disease transmission from donor to recipient, and to mitigate this risk, cortical bone allografts are typically sterilized with high-ionizing gamma radiation (Singh et al. 2016; Nguyen et al. 2007a). The recommended radiation sterilization dose required for eliminating microorganisms from bone grafts historically has been 25–35 kGy (Akkus et al. 2005; Singh et al. 2016; Nguyen et al. 2007b). Fracture properties of human cortical bone, particularly both notched and unnotched fatigue properties, have been shown to be diminished by this standard radiation sterilization dose (Akkus et al. 2005; Akkus and Rimnac 2001; Mitchell et al. 2004). In an effort to mitigate the radiation induced tissue damage imposed by the standard sterilization dose, lower sterilization doses in the range of 15–25 kGy have been explored as an alternative dose as this range provides an acceptable sterility assurance level for tissue allografts (Nguyen et al. 2007b).

The effects of lower alternative radiation sterilization doses on the fatigue properties (i.e., cyclic loading resistance) of bone tissue had not been quantified until recently (Crocker et al. 2023; Pendleton et al. 2019; Ina et al. 2022). Pendleton et al. found that mouse vertebrae irradiated at 17 kGy had a reduced cyclic fatigue life under axial compression compared to 0 kGy, so much so that the monotonic strength at 17 kGy was lower than the prescribed cyclic loading force, and specimens failed after one cycle (Pendleton et al. 2019). In our previous work, we have recently shown that the high-cycle fatigue life under rotating bending (Ina et al. 2022) and the fatigue crack propagation resistance of human cortical bone (Crocker et al. 2023) are radiation dose-dependent, such that higher doses exhibited logarithmically lower fatigue lives and up to a 15-fold reduction in fatigue crack propagation resistance. Accordingly, there appears to be a mechanical benefit to reducing the sterilization dose from the standard dose of 25–35 kGy to extend the performance lifetime of cortical bone allografts subjected to activities of daily living.

The tissue damage mechanism by which high-ionizing gamma radiation reduces both the notched and unnotched fatigue life of human cortical bone has yet to be fully elucidated. It has been shown that radiation-induced collagen chain fragmentation (i.e. cleavage of collagen molecules into smaller polypeptides) and accumulation of stable collagen crosslinks via non-enzymatic glycation develop in bone irradiated at the standard sterilization dose compared to unirradiated bone (Akkus et al. 2005; Pendleton et al. 2019; Burton et al. 2014; Nguyen et al. 2007a; Karim et al. 2013). It has also been suggested that the combination of radiation-induced collagen chain fragmentation and collagen crosslinking may influence the reduction of static and cyclic fracture properties of cortical bone at the standard sterilization dose (Akkus et al. 2005; Pendleton et al. 2019; Burton et al. 2014; Nguyen et al. 2007a). However, to our knowledge, the degree to which radiation induces collagen chain fragmentation and collagen crosslinking of cortical bone within an intermediate dose range of 0–25 kGy has yet to be explored. The objectives of this study therefore were to determine if radiation sterilization has a dose-dependent effect on collagen chain fragmentation and collagen crosslinking of cortical bone between 0 and 25 kGy.

## Materials and methods

### Experimental design and overview

In our previous study (Crocker et al. 2023), bilateral femoral pairs from two human donors with no known pathologies affecting bone health were obtained from the Musculoskeletal Transplant Foundation. Briefly, donors were a 45-year-old female and a 61-year-old male. Compact tension (CT) specimens were machined from the mid-diaphyses and razor-sharpened per previously established protocols (Crocker et al. 2023; Akkus and Rimnac 2001; American Society for Testing and Materials 2020). Specimens were randomly allocated to four irradiation treatment groups: 0 kGy (control), 10 kGy, 17.5 kGy, and 25 kGy (Crocker et al. 2023). Fatigue crack propagation tests were conducted with n = 2–3 specimens per treatment group according to ASTM standard E-647-15e1 (Crocker et al. 2023; American Society for Testing and Materials 2015). Following fatigue testing of our previous CT specimens, bone collagen was isolated from one specimen from both donors in each treatment group. Collagen chain fragmentation was evaluated by conducting SDS-PAGE on the isolated collagen with n = 5 assays per treatment group for each donor. Non-enzymatic and enzymatic collagen crosslinking were evaluated by conducting a fluorometric assay (Oest and Damron 2014) on the isolated collagen with n = 4 assays per treatment group for each donor.

### Collagen isolation

Collagen was isolated from CT specimens per previously established protocols (Akkus et al. 2005; Campo and Betz 1987; Burton et al. 2014; Rittie 2017). Briefly, bone specimens were pulverized in liquid nitrogen in a Spex freezer mill (Metuchen, NJ). The pulverized tissue was dehydrated in 200 proof ethanol then demineralized at 4C for 10 days under gentle rocking agitation in a solution of 0.5 M EDTA (pH 7.5) containing 5 mM concentrations of the following protease inhibitors: benzamidine hydrochloride, iodoacetamide, and phenylmethylsulfonyl fluoride (Campo and Betz 1987; Rittie 2017). The demineralized solution was centrifuged at 3500 rpm for 10 min at 4C and the precipitate was dialyzed (3.5 kDa MW cutoff) against Milli-Q water at 4C for 3 days to remove mineral ions from the solution. The demineralized tissue was then digested for 48 hr in 0.5 M acetic acid (100 ml per 1 g of tissue) with pepsin at 4C such that the ratio of bone tissue to pepsin was 10 to 1 by weight (Akkus et al. 2005). Collagen was then precipitated from the digest solution at 4C for 24 hr with 2.0 M NaCl under gentle rocking agitation, and the precipitate was redissolved in 5 ml of 0.5 M acetic acid and dialyzed (3.5 kDa MW cutoff) against 0.2 M acetic acid to remove salt ions from the solution (Akkus et al. 2005). Following collagen isolation, a Sirius Red Total Collagen Detection Assay (Chondrex, Woodinville, WA) was conducted to determine the concentration of bone collagen within each acid-soluble digest solution. A total of eight acid-soluble collagen digest solutions were attained (one solution for each radiation treatment group from each of the two donors). The acid-soluble collagen solutions were then stored at -20C until chain fragmentation and crosslinking analyses were conducted.

### Collagen chain fragmentation analysis

Collagen solutions and a rat tail collagen standard (Sigma-Aldrich, St. Louis, MO) were lyophilized overnight. Lamaelli buffer (Bio-Rad, Hercules, CA) was added to the lyophilized collagens to attain a concentration of 5 ug of collagen per ul of lamaelli buffer. The collagen samples were then denatured in a 95C wet bath for 2 min. Collagen samples, the rat tail collagen standard, and a Precision Plus Protein Dual Color molecular weight ladder (Bio-Rad, Hercules, CA) were loaded on 4–15% gradient Mini PROTEAN TGX precast SDS-PAGE gels (Bio-Rad, Hercules, CA) (n = 5 assays per treatment group for each donor). The molecular weight ladder and the rat tail collagen standard were loaded at 5 ul per well, and the human bone collagens were loaded at 12 ul per well. SDS-PAGE was conducted at 100 V for 75 min, assuring that the proteins reached the bottom of the gel.

Following electrophoresis, gels were placed in a bath and rinsed in a solution of 50% methanol, 40% ultrapure water, and 10% acetic acid by volume for 30 min under gentle rocking agitation. Gels were then stained with Coomassie blue in a bath for 2 hr under gentle rocking agitation and rinsed with ultrapure water to remove excess stain. The gels were then placed in a bath of destaining solution consisting of 90% ultrapure water, 5% acetic acid, and 5% methanol for 24 hr under gentle rocking agitation, changing the destaining solution once within this period (Burton et al. 2014). Following destaining, gels were transferred to a white light transilluminator and SmartDoc gel imaging enclosure system (Transilluminators, Atkinson, NH), and an image was obtained using a mobile device. Gel images were then analyzed using ImageJ software where optical density profiles of each gel were generated and the baseline of each profile was subtracted. To obtain the degree of radiation-induced collagen fragmentation for irradiated groups, for each gel, first the difference between the area under the gamma, beta, alpha-I and alpha-II optical density peaks at 0 kGy and the area of the total optical density profile at 0 kGy was calculated, thus providing a control condition at 0 kGy of the percentage of molecular weights outside of the known protein chains (F_C_) (Eq. 1). Next, similarly for each irradiated group gel lane, the difference between the area under the gamma, beta, alpha-I and alpha-II optical density peaks and the area of the total optical density profile was calculated, providing a percentage of molecular weights outside of the known protein chains (F_I_) (Eq. 2). The percentage of radiation-induced chain fragmentation at each radiation dose was then calculated as the difference between F_I_ and F_C_, thus indicating a radiation-induced increase in collagen chain fragmentation relative to the 0 kGy control (F_I,C_) (Eq. 3).

1 $$\mathrm{F_{C}}= (\mathrm{A_{Total}}-\mathrm{A}_\gamma -\mathrm{A}_\beta-\mathrm{A}_{\alpha \mathrm{I}}-\mathrm{A}_{\alpha \mathrm{II}})/ \mathrm{A}_{\mathrm{Total \, at \, 0 \, kGy}}$$

2 $$\mathrm{FI }= ({\text{A}_{\text{Total} }-{ \text{A}_{\gamma} }-{ \text{A}_{\beta }}-{ \text{A}_{\alpha\text{I}}}-{ \text{A}_{\alpha \text{II}}}})/{ \text{A}_{\text{Total\, at\, irradiated\, dose}}}$$

3 $$ {\text{FI}},{\text{C }} = {\text{ FI }}{-}{\text{ FC}} $$

### Collagen crosslinking analysis

The extent of non-enzymatic and enzymatic collagen crosslinking was determined by autofluorescence of the acid-soluble collagen digest using a Tecan Infinite M200 plate reader (Research Triangle Park, NC) (Oest and Damron 2014). Isolated collagen solutions from each donor at each radiation dose level were loaded into their own wells, and four readings were conducted per well. Three glycation products were quantified based on their excitation and emission wavelengths: pentosidine (excitation/emission λ 335/385) and non-specific AGEs 1 and 2 (370/440 and 335/400) (all non-enzymatic crosslinks), and pyridinoline (297/395) (an enzymatic crosslink) (Oest and Damron 2014; Vashishth 2009; Vashishth et al. 2001). Because pentosidine is known to be present at low concentrations of one crosslink per 200–300 collagen molecules in unirradiated bone (Vashishth 2009), pentosidine was normalized to a quinine sulfate standard (Oest and Damron 2014; Vashishth et al. 2001). Crosslinking results are expressed in arbitrary units, with the exception of pentosidine, which is expressed in pg of quinine sulfate in relation to the quinine sulfate standard (Oest and Damron 2014). All crosslinking quantities are normalized to collagen concentration of the respective digest sample.

### Statistical analysis

Differences in radiation-induced collagen chain fragmentation and collagen crosslinking with respect to radiation dose level for each donor were assessed using one-way ANOVA (α = 0.05). Tukey’s tests were used to compare radiation-induced collagen chain fragmentation between 0 kGy and all irradiated groups, as well as between 10 and 17.5 kGy, and between 17.5 and 25 kGy for each donor. Dunnett’s tests were used to compare collagen crosslinking between 0 kGy and all other treatment groups for each donor. Significance was taken as *p* < 0.05.

## Results

### Collagen fragmentation

**Fig. 1:**
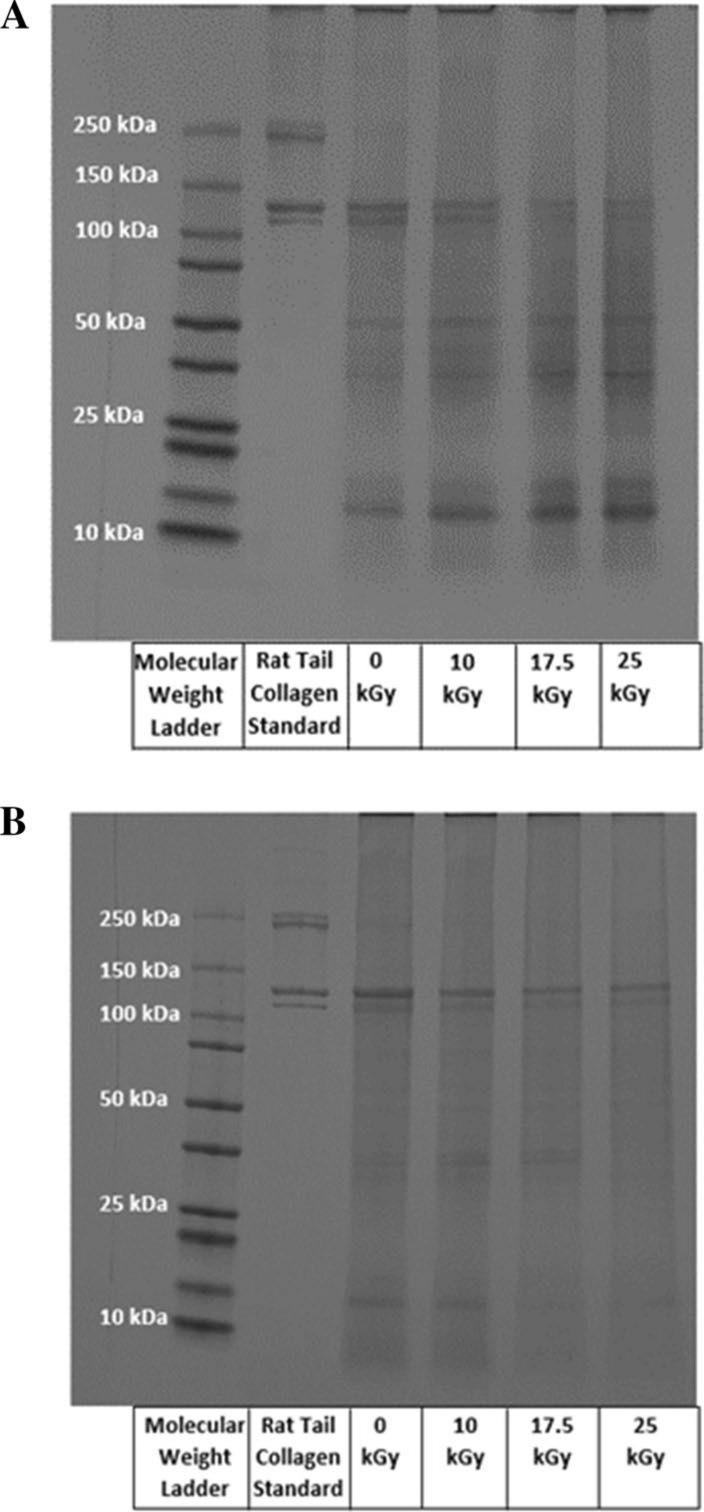
**A** An SDS-PAGE gel of isolated cortical bone collagen from the 45-year-old female donor at each radiation dose level from 0 to 25 kGy. Gamma, beta, alpha-I, and alpha-II chains are identified in the rat tail collagen standard lane at approximately 300 kDa, 240 kDa, 130 kDa, and 120 kDa, respectively. **B** An SDS-PAGE gel of isolated cortical bone collagen from the 61-year-old male donor at each radiation dose level from 0 to 25 kGy. Gamma, beta, alpha-I, and alpha-II chains are identified in the rat tail collagen standard lane at approximately 300 kDa, 240 kDa, 130 kDa, and 120 kDa, respectively

**Fig. 2:**
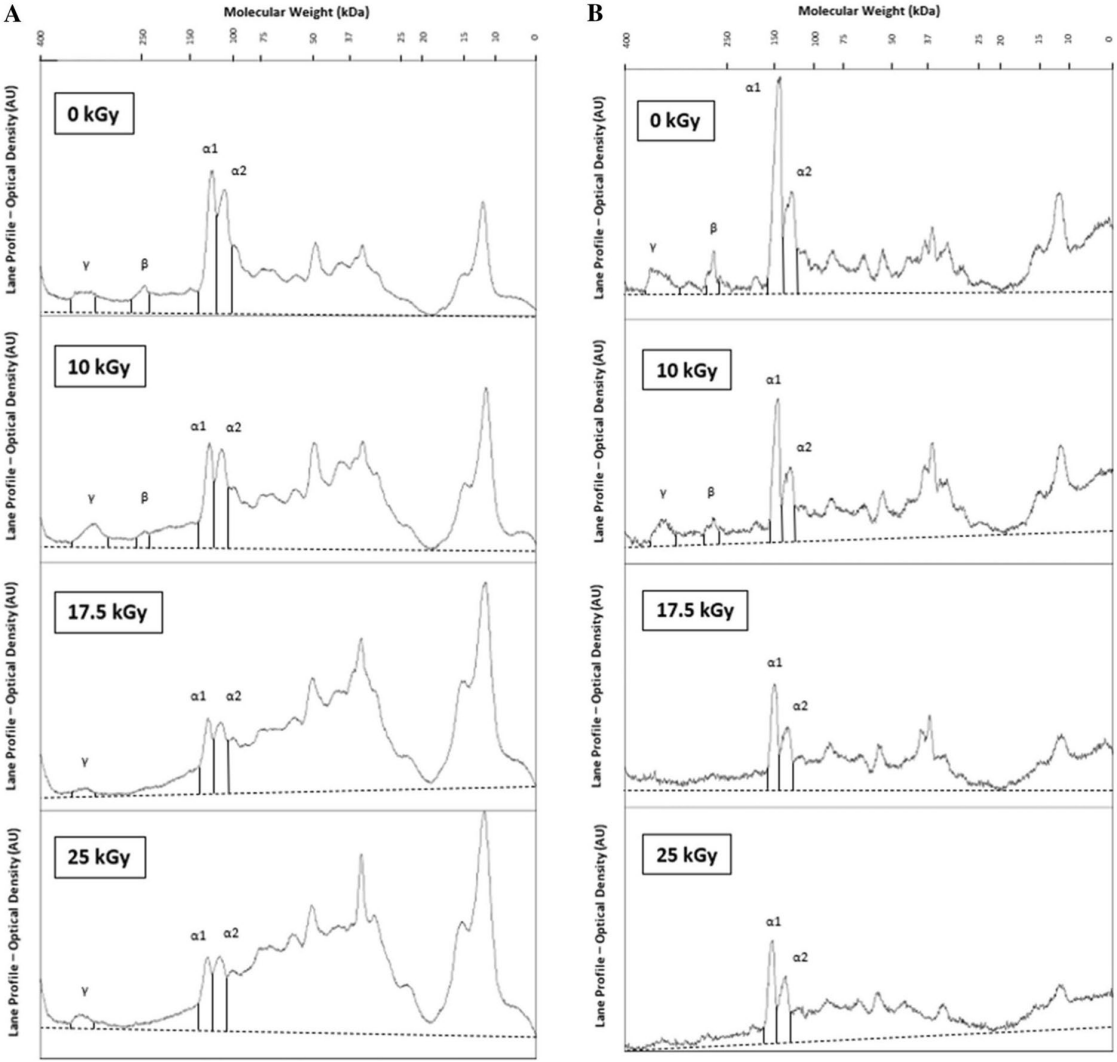
**A** ImageJ optical density spectra of each lane of a gel from the 45-year-old female donor in Fig. 1A. The dashed line represents baseline subtraction prior to total spectrum and protein chain area calculations (enclosed). **B** ImageJ optical density spectra of each lane of a gel from the 61-year-old male donor in Fig. 1B. The dashed line represents baseline subtraction prior to total spectrum and protein chain area calculations (enclosed)

SDS-PAGE gels and their optical density profiles identified alpha-I, alpha-II, beta, and gamma protein chains within the collagen matrix at 0 kGy (Figs. 1 and 2). The optical density of each of these protein chains appeared to decrease with increasing radiation dose. Alpha-I and alpha-II protein chains remained identifiable at each radiation dose up to 25 kGy for both donors. Beta protein chains were not identifiable at radiation doses above 10 kGy for both donors. Gamma protein chains also remained identifiable at each radiation dose up to 25 kGy for the 45-year-old female donor; however, the gamma protein chains were not identifiable at 17.5 kGy at radiation doses above 10 kGy for the 61-year-old male donor. Lower molecular weight protein fragments became more prevalent with increasing radiation dose as demonstrated by a broader distribution of molecular weights across the length of the gels, particularly at molecular weights below the alpha-I and alpha-II chains, often referred as “smearing” of the gels, for both donors. Additionally, this phenomenon is shown in the optical density profiles of the gels where the optical densities of molecular weights not identified as alpha-I, alpha-II, beta, or gamma chains progressively increases with increasing radiation dose (Fig. 2). Collectively, a qualitative visualization of the SDS-PAGE gels and optical density profiles indicated that the collagen molecular structure was increasingly disrupted by cleavage of collagen molecules with increasing radiation dose. The observable patterns of the gels and optical density profiles described above were consistent for each of the five gels within each donor.

**Fig. 3:**
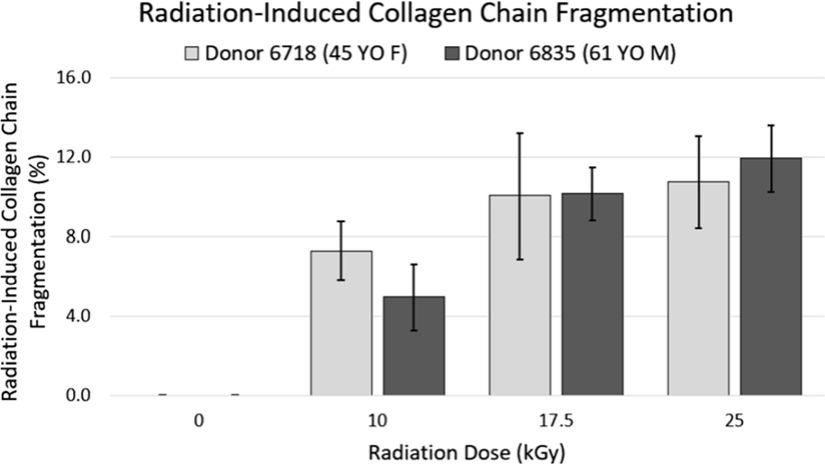
Mean and standard deviation of radiation-induced collagen chain fragmentation (n = 5) for the two donors. One-way ANOVA indicated a dose-dependent increase in radiation-induced collagen chain fragmentation with increasing radiation dose for each donor (*p* < 0.001)

For the 45-year-old female donor, collagen chain fragmentation increased in a dose-dependent manner (*p* < 0.001) (Fig. 3). The collagen chain fragmentation at each irradiated treatment group relative to the 0 kGy control was: 7.29 ± 1.62% at 10 kGy, 10.04 ± 3.54% at 17.5 kGy, and 10.73 ± 2.57% at 25 kGy. More collagen chain fragmentation was observed at all irradiated treatment groups relative to the 0 kGy control (*p* ≤ 0.001). No difference in collagen chain fragmentation was observed between 10 and 17.5 kGy (*p* = 0.28), or between 17.5 and 25 kGy (*p* = 0.97).

For the 61-year-old male donor, collagen chain fragmentation also increased in a dose-dependent manner (*p* < 0.001) (Fig. 3). The collagen chain fragmentation at each irradiated treatment group relative to the 0 kGy control was: 4.97 ± 1.88% at 10 kGy, 10.14 ± 1.48% at 17.5 kGy, and 11.91 ± 1.88% at 25 kGy. More collagen chain fragmentation was observed at all irradiated treatment groups relative to the 0 kGy control (*p* ≤ 0.001). More collagen chain fragmentation was observed at 17.5 kGy than 10 kGy (*p* < 0.001). No difference in collagen chain fragmentation was observed between 17.5 and 25 kGy (*p* = 0.29).

### Collagen crosslinking

**Fig. 4:**
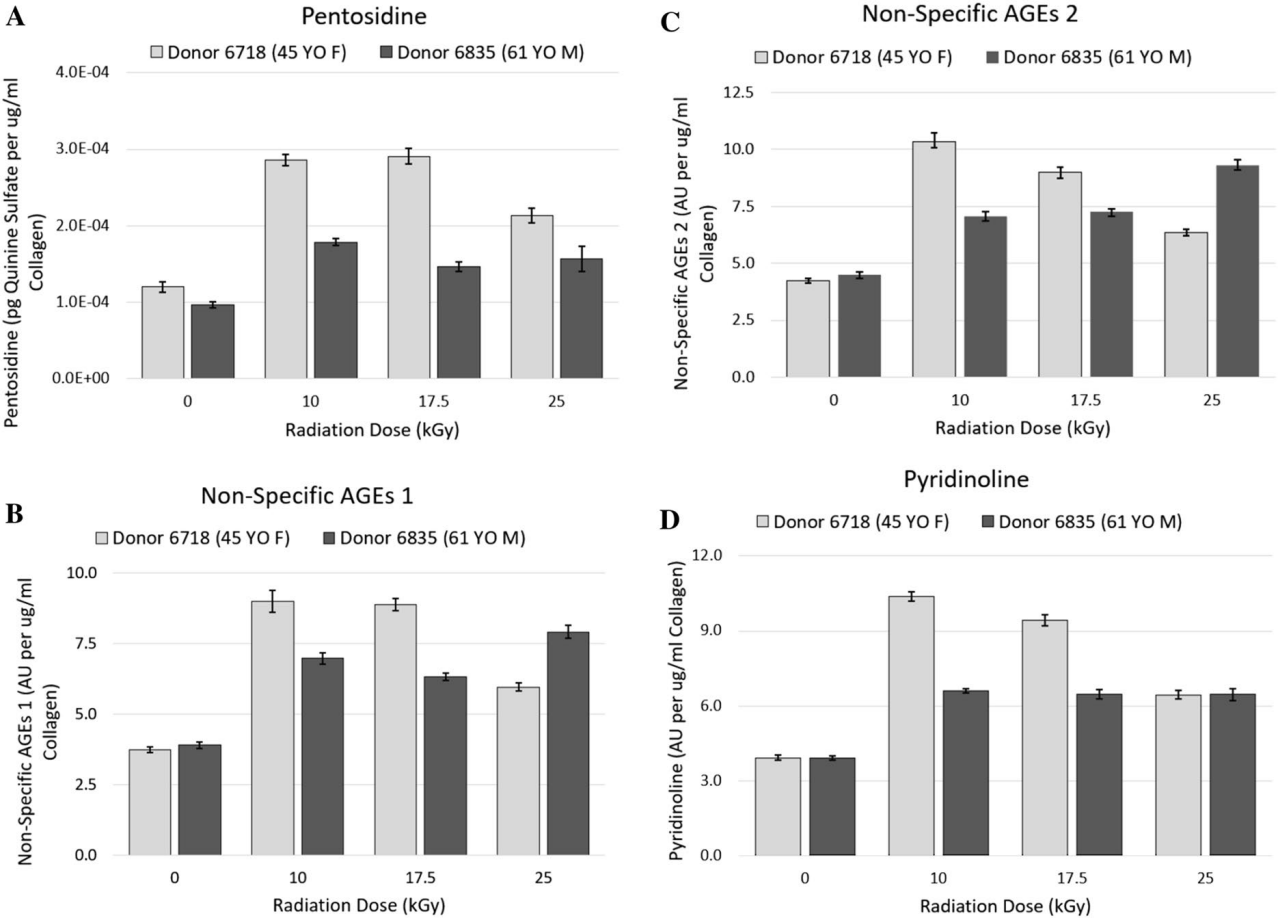
**A** Mean and standard deviation of pentosidine, a non-enzymatic crosslink (n = 4), for both donors, normalized to collagen concentration of the digest solution. For both donors, pentosidine increased from 0 to 25 kGy but did not demonstrate dose-dependency (*p* < 0.001). More pentosidine was observed at all irradiated groups relative to the 0 kGy control group (*p* < 0.001). **B** Mean and standard deviation of non-specific AGEs1, non-enzymatic crosslinks (n = 4), for both donors, normalized to collagen concentration of the digest solution. For both donors, non-specific AGEs 1 increased from 0 to 25 kGy but did not demonstrate dose-dependency (*p* < 0.001). More non-specific AGEs 1 were observed at all irradiated groups relative to the 0 kGy control group (*p* < 0.001). **C** Mean and standard deviation of non-specific AGEs 2, non-enzymatic crosslinks (n = 4), for both donors, normalized to collagen concentration of the digest solution. For both donors, non-specific AGEs 2 increased from 0 to 25 kGy but did not demonstrate dose-dependency (*p* < 0.001). More non-specific AGEs 2 were observed at all irradiated groups relative to the 0 kGy control group (*p* < 0.001). **D** Mean and standard deviation of pyridinoline, an enzymatic crosslink (n = 4), for both donors, normalized to collagen concentration of the digest solution. For both donors, pyridinoline increased from 0 to 25 kGy but did not demonstrate dose-dependency (*p* < 0.001). More pyridinoline was observed at all irradiated groups relative to the 0 kGy control group (*p* < 0.001)

For the 45-year-old female donor, pentosidine increased from 0 to 17.5 kGy, and then decreased from 17.5 to 25 kGy (*p* < 0.001) (Fig. 4A). Non-specific AGEs 1 increased from 0 to 10 kGy, and then decreased from 10 to 17.5 kGy before increasing again from 17.5 to 25 kGy (*p* < 0.001) (Fig. 4B). Non-specific AGEs 2 increased dose-dependently from 0 to 25 kGy (*p* < 0.001) (Fig. 4C). Pyridinoline increased from 0 to 10 kGy and then decreased from 10 to 25 kGy (*p* < 0.001) (Fig. 4D). More pentosidine, non-specific AGEs 1 and 2, and pyridinoline were observed at all irradiated groups relative to the 0 kGy control group (*p* < 0.001).

For the 61-year-old male donor, pentosidine increased from 0 to 17.5 kGy and then decreased from 17.5 to 25 kGy (*p* < 0.001) (Fig. 4A). Non-specific AGEs 1 and 2, and pyridinoline, increased from 0 to 10 kGy and then decreased from 10 to 25 kGy (*p* < 0.001) (Fig. 4B–D). More pentosidine, non-specific AGEs 1 and 2, and pyridinoline were observed at all irradiated groups relative to the 0 kGy control group (*p* < 0.001).

## Discussion

We observed an increase in radiation-induced collagen chain fragmentation from 0 kGy to a clinically standard dose of 25 kGy (Fig. 3). This is consistent with the work of others (Akkus et al. 2005; Burton et al. 2014). Additionally, we observed a dose-dependent increase in radiation-induced collagen chain fragmentation within this dose range (Fig. 3). This is consistent with the findings of Pendleton et al. (2019) for the dose-dependent effects of irradiation on the collagen matrix of trabecular mouse bone. When collagen is irradiated, hydroxyl radicals are formed through ionization of water molecules in the organic matrix (Akkus et al. 2005). These hydroxyl radicals have been speculated to be responsible for cleaving the collagen molecules into smaller peptides, specifically at proline and hydroxyproline residues (Monboisse and Borel 1992; Nguyen et al. 2005), thus suggesting hydroxyl radical formation as the mechanism behind radiation-induced collagen chain fragmentation. Akkus et al. (2005) tested this theory by treating cortical bone specimens with a thiourea free radical scavenging treatment prior to irradiation. Thiourea undergoes a direct chemical reaction with hydroxyl radicals during ionizing radiation, reducing the effects of the free radicals on the collagen molecular structure (Akkus et al. 2005). Not surprisingly, Akkus et al. (2005) found that cortical bone treated with the free radical scavenger prior to irradiation underwent less collagen chain fragmentation than cortical bone irradiated without the free radical scavenger treatment, as assessed qualitatively by SDS-PAGE. Furthermore, others have reported that the amount of free radical formation increases linearly with radiation dose in the dose range of 0–200 Gy, a range that is associated with cancer therapies (Nguyen et al. 2005; Monboisse et al. 1988). However, to our knowledge, the dose-dependency of free radical formation within a clinically relevant dose-range for allograft sterilization has not been studied. Taken together, we believe that radiation-induced hydroxyl free radical formation is responsible for collagen chain fragmentation within this dose range of 0–25 kGy. We observed a non-linear increase in radiation-induced chain fragmentation, and this suggests that free radical formation may reach a plateau at a dose level within this range, likely dependent on the amount of water available in the organic matrix per donor source (Nyman et al. 2013; Granke et al. 2015).

We observed an increase in the amount of pentosidine (Fig. 4A), non-specific AGEs 1 (Fig. 4B), non-specific AGEs 2 (Fig. 4C), and pyridinoline (Fig. 4D) between 0 kGy and all irradiated conditions (*p* < 0.001). This is consistent with what others have found (Pendleton et al. 2019; Oest and Damron 2014). Specifically, pyridinolines are mature (non-reducible) divalent crosslinks that form interfibrillar covalent bonds at hydroxylysine and lysine residues by the action of the lysyl hydroxylase enzyme (Di Medio and Brandi 2021; Hernandez et al. 2005). Advanced glycation end products on the other hand are covalent bonds of oxidizing sugars and free amino acid groups of collagen proteins, found both between and within fibrils (Di Medio and Brandi 2021). Our crosslinking data suggests that any amount of radiation between 10 and 25 kGy exposes free amino acid groups on collagen proteins, initiating mature crosslinking at free amino acid residues, both through glycation and covalent bonding specifically at lysines and hydroxylysines. Furthermore, our data suggests that the amount of radiation-induced crosslinking is limited by the amount of oxidizing sugars (Fu et al. 1994) and lysyl hydroxylase availability within the organic matrix, as demonstrated by the lack of crosslink accumulation with increasing radiation dose.

In our previous work, we showed that fatigue crack propagation resistance of these same specimens decreased with increasing radiation dose (Crocker et al. 2023). Specifically, specimens irradiated at 10 kGy were less resistant to fatigue crack propagation than the 0 kGy control condition, specimens irradiated at 17.5 kGy were less resistant to fatigue crack propagation than those irradiated at 10 kGy, and there was no detectable difference in fatigue crack propagation resistance between 17.5 and 25 kGy (Crocker et al. 2023). Increases in chain fragmentation between dose levels evaluated in the current study coincide with corresponding losses in fatigue crack propagation resistance between dose levels in our previous study (Crocker et al. 2023), suggesting that radiation-induced collagen chain fragmentation is an influential mechanism in notched fatigue resistance loss resulting from irradiation. For the 61-year-old male donor, the plateau in chain fragmentation at 17.5 kGy corresponds to a similar pattern with our previous fatigue crack propagation resistance findings in that there was no additional loss in fracture resistance above 17.5 kGy. While the 45-year-old female donor showed a plateau in chain fragmentation at 10 kGy rather than at 17.5 kGy, the 7% increase in fragmentation from 0 to 10 kGy suggests that collagen from this donor may have been more sensitive to lower doses of radiation. Taking together the current study and our previous work (Crocker et al. 2023), it appears that any amount of irradiation at 10 kGy or higher induces collagen chain fragmentation, and once chain fragmentation is initiated, notched fatigue crack propagation resistance is diminished.

Crosslink accumulation likely also contributes to the radiation-induced loss in fatigue crack propagation resistance. Inter- and intra-fibrillar crosslink accumulation plays an important role in both intrinsic and extrinsic toughening mechanisms of cortical bone tissue; thus, understanding their behavior is an important consideration in interpreting fatigue crack propagation resistance of cortical bone tissue (Barth et al. 2011). Intra-fibrillar crosslink formation influences plasticity via molecular uncoiling and intermolecular sliding (Barth et al. 2011). An increase in intra-fibrillar crosslinking thus decreases plasticity by limiting these mechanisms, thus stiffening the bone tissue (Barth et al. 2011). More importantly for fatigue crack propagation resistance, extrinsic toughening mechanisms that blunt crack growth such as crack-tip shielding, crack deflection, and ligament bridging are hindered by an accumulation of inter-fibrillar crosslink formation as the tissue’s ability to dissipate energy is compromised (Barth et al. 2011; Ritchie et al. 2008). As such, our data suggests that 10 kGy may be a sufficient dose of radiation to hinder extrinsic toughening mechanisms in cortical bone tissue that retard crack growth. However, with no further crosslink accumulation with increasing radiation dose, crosslinking alone cannot account for further reduction in fatigue crack propagation resistance with increasing radiation dose (Pendleton et al. 2021). Thus, our data suggests that collagen chain fragmentation is the principle molecular mechanism responsible for the dose-dependency of radiation-induced losses in fatigue crack propagation resistance of cortical bone irradiated with clinically relevant doses ranging from 10 to 25 kGy, and preserving the notched fatigue life of cortical bone likely requires an interruption of the pathway by which radiation compromises the integrity of the collagen structure by cleaving collagen molecules into smaller polypeptides through free radical formation.

This study had limitations. First, cortical bone samples used to obtain the collagen digest solutions were not machined from the same anatomical location, potentially introducing baseline differences in collagen crosslinking between samples prior to irradiation (Paschalis et al. 2003). However, with Dunnett’s tests supporting an increase in crosslinking at all irradiation doses relative to the control condition, baseline crosslinking conditions likely have little influence on dose-to-dose effects. Additionally, the crosslinking data obtained from the fluorometric assay may be influenced by non-collagenous proteins within the organic matrix and crosslink types whose excitation/emission wavelengths overlap with those of the target crosslink. The assay does not explicitly identify collagen, nor the target crosslinks, as the source of fluorescence (Vashishth 2009). However, collagen comprises about 90% of the organic matrix of cortical bone, thus the influence of non-collagenous proteins and crosslinks on fluorescence measurements should be minimal. Lastly, collagen donor sources had slightly different sensitivity to irradiation. Exploring the influence of irradiation on collagen chain fragmentation and crosslinking on additional donors may be a potential future avenue to provide a better understanding of how collagen characteristics are influenced by irradiation for the broader population.

## Conclusions

Our findings support that radiation-induced collagen chain fragmentation of cortical bone is radiation dose-dependent within a clinically relevant dose range of 0–25 kGy, whereas the crosslinks examined did not increase in a dose-dependent manner. Radiation-induced collagen chain fragmentation is likely to be the main cause of dose-dependent losses in fatigue crack propagation resistance of cortical bone given that increases in collagen chain fragmentation coincide with reductions in fatigue crack propagation resistance. Crosslinking may also contribute to radiation-induced reductions in fatigue crack propagation with any amount of radiation at or above 10 kGy by influencing the extrinsic toughening mechanisms of cortical bone that blunt crack growth, such as crack-tip shielding and ligament bridging, but crosslinking alone does not explain the radiation dose-dependence of notched fatigue resistance. While the standard radiation dose for sterilization of cortical bone tissue historically has been allocated as 25 kGy or higher, radiation doses in the range of 10–15 kGy have been shown to provide acceptable sterility assurance (Nguyen et al. 2007b, 2013). Given the radiation dose-dependency of collagen chain fragmentation and its detrimental effect on notched fatigue resistance, decreasing the sterilization radiation dose to the lowest effective dose could improve high-cycle and notched fatigue resistance of sterilized cortical bone allografts by mitigating the accumulation of breakage of collagen molecules into smaller polypeptides. Further studies to examine the dose-dependent effects of radiation-induced collagen chain fragmentation and crosslinking on fatigue resistance within a lower dose range of 0–15 kGy may be valuable. Understanding the dose-dependency of these biological mechanisms and their relationship to fatigue life within a lower dose range could potentially provide evidence to support novel sterilization methods and suggested doses for sterilization to both provide acceptable sterility and extend the fatigue life of cortical bone allografts.

## Data Availability

No datasets were generated or analysed during the current study.
